# Novel ALK gene mutation in inflammatory myofibroblastic tumor of the thyroid: a case report

**DOI:** 10.3389/fonc.2025.1616075

**Published:** 2025-06-25

**Authors:** Yang Guangxu, Li Yao, Xie Jing, Liu Hongsheng

**Affiliations:** ^1^ Department of Ultrasonography, The Affiliated Hospital of Zunyi Medical University, Zunyi, Guizhou, China; ^2^ Department of Pathology, The Affiliated Hospital of Zunyi Medical University, Zunyi, Guizhou, China; ^3^ Department of Pathology, Rui-jin Hospital, Shanghai Jiao-tong University School of Medicine, Shanghai, China; ^4^ Department of Radiology, Guangzhou Women and Children’s Medical Center, Guangzhou Medical University, Guangdong Provincial Clinical Research Center for Child Health, Guangzhou, China

**Keywords:** inflammatory myofibroblastic tumor, thyroid, ALK-positive, gene mutation, case report

## Abstract

**Background:**

Inflammatory myofibroblastic tumor (IMT) is a rare soft tissue neoplasm, exceptionally uncommon in the thyroid. Approximately 50%–70% of IMT cases exhibit ALK gene rearrangements or fusions, while ALK point mutations are rare. We report a novel ALK gene mutation, ALK-R395H, in a case of thyroid IMT and review the relevant literature.

**Case report:**

A 43-year-old female patient presented with a thyroid mass discovered two months prior. Ultrasound revealed a solid hypoechoic mass in the middle of the left thyroid lobe. Histopathology showed characteristic spindle cell proliferation with plasma cell and lymphocyte infiltration. Immunohistochemistry demonstrated strong expression of Vimentin and ALK-1 in spindle cells, focal SMA expression, and strong positivity for Galectin-3, PAX-8, and TTF-1. Next-generation sequencing identified mutations in NTRK1, GNAS, RB1, and ALK, with a G1184A mutation in ALK exon 5, resulting in a missense mutation ALK(p.R395H) in the extracellular domain, the function of which remains to be elucidated. The pathological diagnosis was thyroid IMT; however, strong expression of thyroid epithelial markers and the ALK mutation suggested possible thyroid carcinoma components or malignant potential. The patient underwent left thyroid lobectomy with isthmus resection, received no adjuvant therapy, and showed no recurrence after 37 months of follow-up.

**Conclusion:**

This case reports the discovery of the ALK-R395H mutation in thyroid IMT, providing new insights into its molecular characteristics.

## 1 Introduction

Inflammatory myofibroblastic tumor (IMT) is a rare, low-grade malignant or borderline mesenchymal neoplasm (1). It predominantly affects children and adolescents and can arise in any anatomical site, with the lungs, abdomen, and soft tissues being the most common locations, while its occurrence in the thyroid gland is exceedingly rare. The ALK gene, which encodes a receptor tyrosine kinase, is implicated in various cancers and may promote tumorigenesis and progression through mutations or fusions (2). Approximately 50%-70% of IMT cases exhibit ALK gene rearrangements or fusions, and about 50%-60% show positive ALK protein expression (3), whereas point mutations in the ALK gene are relatively uncommon. In this case report, a 43-year-old female patient was found, for the first time via next-generation sequencing (NGS), to harbor mutations in four genes within the lesion: NTRK1, GNAS, RB1, and ALK. The NTRK1 and GNAS mutations were located in non-coding regions, and the RB1 (p.S114L) missense mutation was classified as a non-hotspot mutation. Notably, a novel mutation, G1184A, was identified in exon 5 of the ALK gene, resulting in the missense mutation ALK (p.R395H), which substitutes arginine with histidine at position 395 of the encoded protein. While ALK mutations are typically associated with benign or low malignant potential in IMT (4, 5), the mutation in this case is situated in a cancer hotspot region, suggesting a potential risk of malignant transformation. Furthermore, the strong positive expression of Galectin-3, PAX-8, and TTF-1 indicates a possible complex relationship between IMT and thyroid epithelial tumors. This is the first report of the ALK-R395H mutation in thyroid IMT, accompanied by a review of the relevant literature (**Table 1**) (6–12). This finding broadens the genetic landscape of IMT, offers new insights into its molecular characteristics, and may inform future diagnostic and therapeutic approaches.

**Table 1:** List of formerly reported IMT cases.

Author and year	Age (yr)/sex	Clinical manifestation	Ultrasonic manifestation	Immunohistochemistry (ALK, SMA, MSA and Vim)	Treatment	Follow-up
Trimeche,2009 (6)	18/emale	3.0 cm painless right thyroid mass	Hyperechoic nodule with numerous calcifications	Positive: Vim, SMA Negative: ALK-1	Subtotal thyroidectomy	No recurrence 9 months after surgery
Kim,2014 (7)	50/female	0.6 cm right thyroid mass	lobulated hypoechoic mass in the thyroid	Positive: SMA Negative: ALK-1	Total thyroidectomy	Doing well 1 year after surgery
Marylilly,2016 (8)	61/male	Painless right thyroid lobe mass	Hypoechoic right thyroid nodule with cystic degeneration	Positive: SMA,ALK-1,Vim	Total thyroidectomy	Doing well 1 year after surgery
Duan,2017 (9)	57/male	Painless thyroid lobe mass	Hypoechoic left thyroid lobe mass	Positive: ALK-1, SMA,Vim	Subtotal thyroidectomy + radiation therapy + steroid therapy	Alive with recurrence and relapse
Zhang,2017 (10)	64/female	4.0 cm painless mass in the left thyroid lobe	Hypoechoic nodule in the left thyroid lobe	Positive: Vim, SMA Negative: ALK-1	Left subtotal thyroidectomy plus right partial thyroidectomy	No recurrence 4 months after surgery
An, 2018 (11)	12/Male	3.5-cm tender mass in the left thyroid lobe	Irregular, sheet-like hypoechoic area with punctate calcifications	Positive: Vim, SMA Negative: ALK	Left thyroidectomy+ banded muscle +part of sternocleidomastoid muscle	No recurrence during the 4-year follow-up
Li,2019 (12)	34/female	4.0 cm painless left thyroid mass	Hypoechoic left thyroid lobe mass	Positive: ALK-1, SMA, Vim	Left thyroidectomy	Alive without recurrence 10 months after surgery

## 2 Case presentation

**Figure 1:**
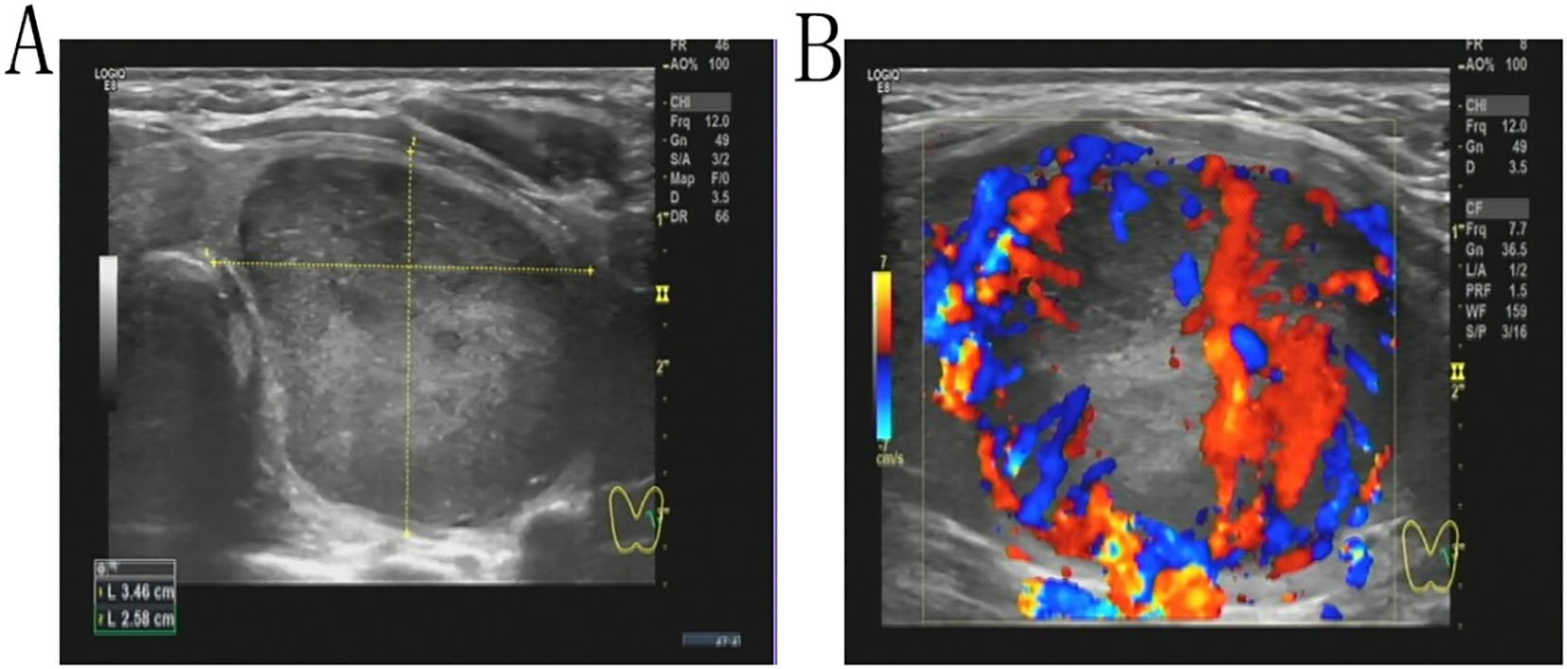
A solid hypoechoic mass measuring 3.46 cm × 2.58 cm × 4.55 cm is present in the midportion of the left thyroid lobe **(A)**. Color Doppler Flow Imaging (CDFI) demonstrates increased vascularity within the mass **(B)**.

**Figure 2:**
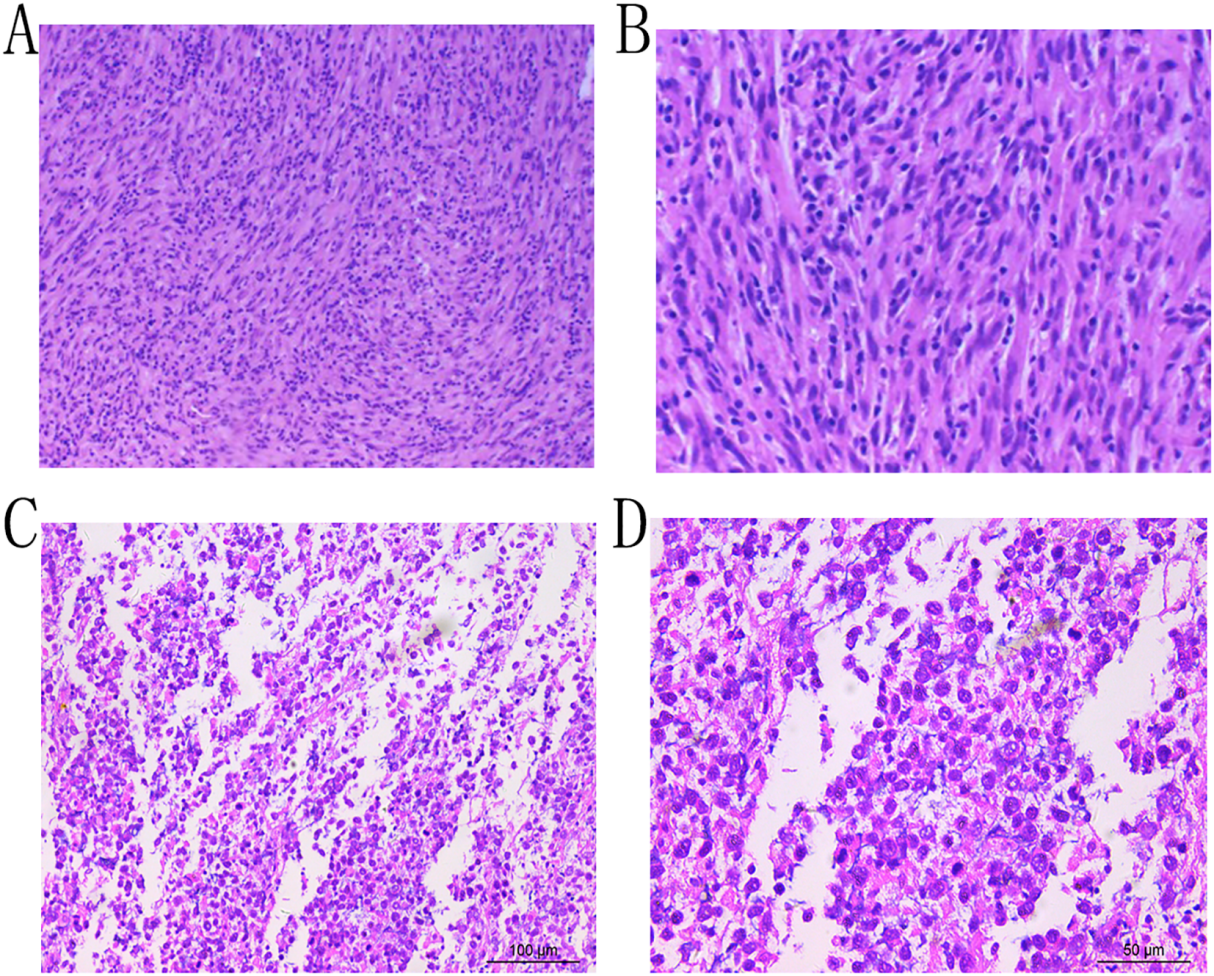
Microscopic examination reveals proliferation of spindle cells arranged in fascicles, exhibiting nuclear pleomorphism, with admixed plasma cells and lymphocytes (Hematoxylin and Eosin stain, ×10, **A**; ×20, **B**;×20, **C**; ×40, **D**).

**Figure 3:**
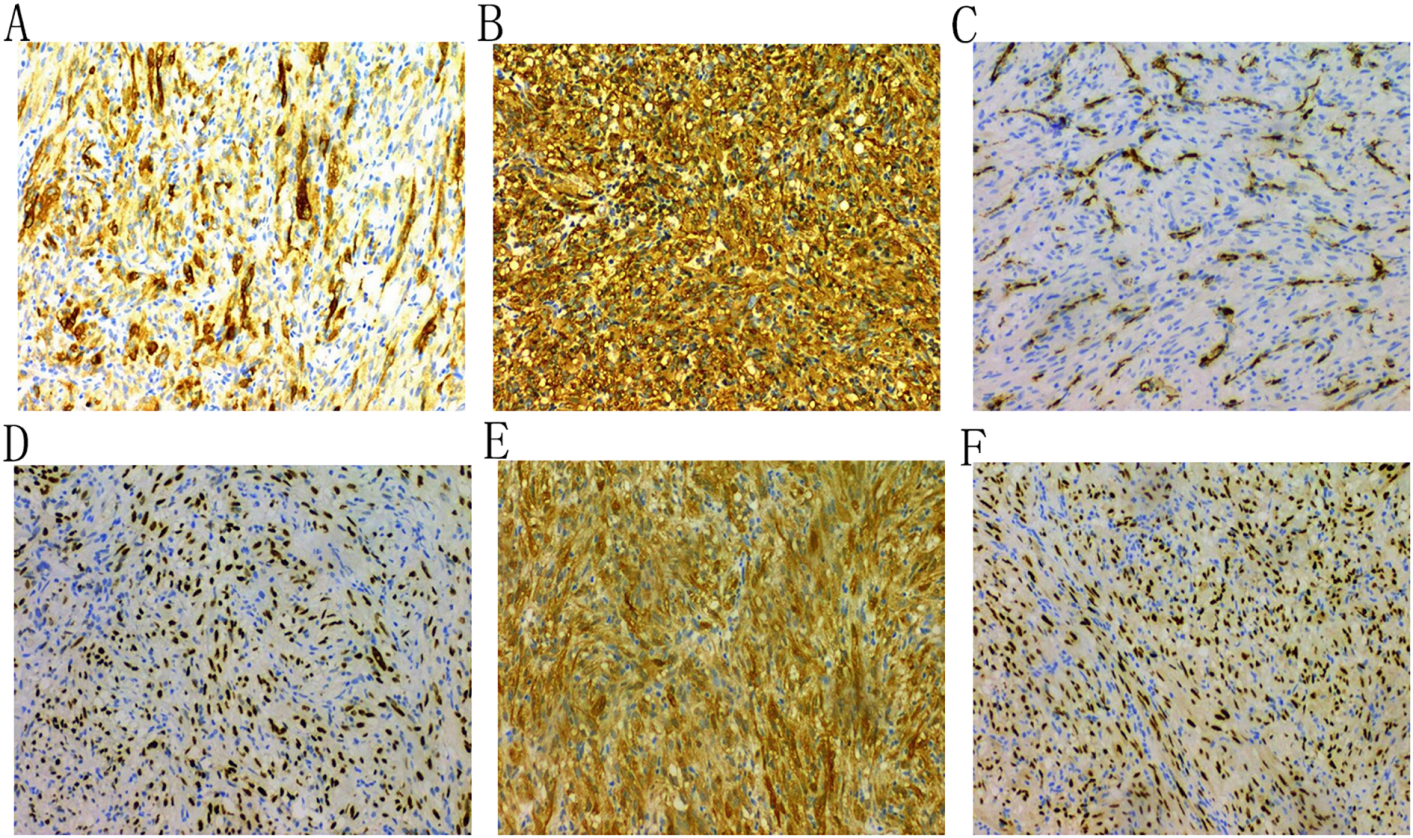
Immunohistochemical analysis shows that the tumor cells are positive for ALK-1 **(A)**, Vimentin **(B)**, and SMA **(C)**; and for TTF-1 **(D)**, Galectin-3 **(E)**, and PAX-8 **(F)**.

A 43-year-old female patient was admitted to the hospital due to the discovery of a neck mass two months prior. Physical examination revealed a palpable oval mass in the left lobe of the thyroid, which was firm and non-tender. Laboratory tests showed that all thyroid function indicators were normal. Ultrasound examination revealed diffusely reduced echogenicity in the bilateral thyroid parenchyma with uneven distribution. In the middle of the left thyroid lobe, there was a 3.46 cm × 2.58 cm × 4.55 cm solid hypoechoic mass (**Figure 1A**). Color Doppler flow imaging (CDFI) showed rich blood supply within the mass (**Figure 1B**). No enlarged lymph nodes were observed in the bilateral neck. The ultrasound diagnosis was a middle left thyroid lobe solid nodule classified as TI-RADS 4A. The patient underwent ultrasound-guided fine needle aspiration biopsy (FNAB) of the left thyroid lobe mass, and pathology suggested a possible inflammatory proliferative lesion. Subsequently, the patient underwent left lobectomy with isthmusectomy. Intraoperatively, a gray-brown solid mass approximately 5.0 cm in diameter was observed in the middle of the left thyroid lobe, with clear boundaries from the surrounding tissues. Histopathologically, the lesion in the midportion of the left thyroid lobe exhibited typical spindle cell proliferation, with the spindle cells arranged in fascicles. No mitotic figures were observed, and the nuclei showed slight pleomorphism (**Figures 2A, B**). Additionally, the lesion contained an infiltrate of mature inflammatory cells, such as plasma cells and lymphocytes, along with hyaline collagen in the stroma and some cells displaying abundant cytoplasm (**Figures 2C, D**). Immunohistochemical staining was performed on the specimen using the Ventana Ultraview two-step method. Immunohistochemical analysis for CD34, S100, Desmin, Bcl-2, and STAT-6 excluded the possibilities of solitary fibrous tumor and malignant peripheral nerve sheath tumor. Additionally, calcitonin (CT) and Congo red staining were both negative, ruling out medullary thyroid carcinoma. ALK-1 (**Figure 3A**) and Vimentin showed strong expression in the spindle cells (**Figure 3B**). Cytokeratin (CK) and smooth muscle actin (SMA) showed focal expression (**Figure 3C**). Ki-67 was positive in 10% of cells, and CD68 showed partial positivity. However, TTF-1 (**Figure 3D**), Galectin-3 (**Figure 3E**), and PAX-8 (**Figure 3F**) showed strong positive expression. CK19 and mesothelial cell marker (MC) showed partial positive expression, despite thyroglobulin (TG) being negative. This may suggest the concurrence of a thyroid epithelial-origin tumor. To further clarify the nature of the lesion, we performed next-generation sequencing (NGS) on the thyroid lesion using the Illumina platform, targeting 66 thyroid tumor-related genes and 177 gene fusion points (target genes are listed in **Supplementary Tables 1** and **2**, and results in **Supplementary Table 3**). The results revealed mutations in four genes: NTRK1, GNAS, RB1, and ALK. The NTRK1 and GNAS mutations were located in non-coding regions and were suspected to be benign variants. The RB1 (p.S114L) was a missense mutation, classified as a non-hotspot mutation in this case. Additionally, a novel mutation, G1184A, was identified in exon 5 of the ALK gene with a mutation rate of 1.05%, resulting in the missense mutation ALK (p.R395H), which changes arginine to histidine at position 395 of the encoded protein. This mutation is located in a cancer hotspot region. Currently, there are no reports of this mutation in the COSMIC and ClinVar databases. Therefore, based on the immunohistochemical results and morphological features, the diagnosis was thyroid inflammatory myofibroblastic tumor (IMT) with features suggesting possible concurrence of thyroid cancer or malignant potential. However, the patient did not receive adjuvant therapy and was followed up at 8, 16, 30, and 37 months postoperatively. Thyroid function tests showed slightly elevated levels of serum thyroglobulin and anti-thyroglobulin antibodies. Thyroid ultrasound indicated diffuse lesions in the right thyroid lobe, with no enlarged lymph nodes in the bilateral neck and no recurrence of IMT.

## 3 Discussion

Thyroid inflammatory myofibroblastic tumor (IMT) is an extremely rare soft tissue neoplasm characterized by the proliferation of myofibroblasts and fibroblasts, accompanied by varying degrees of infiltration by plasma cells, lymphocytes, and eosinophils. Its etiology and pathogenesis remain unclear but may be associated with inflammatory and immunological abnormalities (13, 14). Due to its rarity, literature on thyroid IMT is limited. A recent retrospective case series involving 17 cases was excluded from this analysis due to insufficient individual patient data (14). Additionally, a case of cervical IMT invading the thyroid was not included (15). Based on our literature review and data presented in **Table 1**, among the 8 reported cases of thyroid IMT, the average age was 42 years (range 12–64 years), with 3 males and 5 females. The most common clinical presentation was a painless neck mass, with imaging typically revealing hypoechoic nodules. Immunohistochemistry showed ALK positivity in 4 cases (50%). Notably, only our case had pathogenic gene mutations confirmed by gene sequencing. All cases underwent thyroid surgery, with 2 receiving postoperative adjuvant therapy.

The ALK gene, located at 2p23.2-p23.1, comprises 29 exons and encodes a protein with extracellular, transmembrane, and kinase domains (16). Tumorigenesis and progression are significantly associated with aberrant alterations in receptor tyrosine kinase genes (17). In IMT, ALK gene rearrangements or fusions occur in approximately 50%-70% of cases (5), with common fusion partners including TPM3, TPM4, SEC31A, TFG, RANBP2, CLTC, FN1, LMNA, and PRKAR1A (16, 18). Notably, RANBP2 is linked to the epithelioid inflammatory myofibroblastic sarcoma subtype (19). While ALK point mutations are rare in IMT and generally associated with benign or low malignant potential, certain mutations can lead to treatment resistance and potentially more aggressive behavior. Olanich et al. (4) reported a case of IMT where the F1174L mutation emerged during crizotinib treatment, leading to treatment resistance and disease progression. The F1174L mutation, located in the ALK kinase domain, enhances ALK phosphorylation, cell growth, and downstream signaling, suggesting that hotspot mutations may increase the risk of malignant transformation. Similarly, Wang et al. (5)noted that mutations like F1174L may cause treatment resistance, necessitating alternative inhibitors. In anaplastic thyroid carcinoma, two ALK point mutations, C3592T and G3602A, were identified in the tyrosine kinase domain, promoting cell proliferation and invasion by activating signaling pathways (20). In our case, the ALK-R395H mutation was located in the extracellular domain of the ALK protein, which is primarily responsible for ligand binding to trigger signal transduction. Although this mutation occurs in a cancer hotspot region, it is not in the kinase domain, and its role in promoting cell proliferation, invasion, or malignant transformation requires further investigation.

Immunohistochemical detection of ALK protein is a critical diagnostic tool for IMT, with ALK gene rearrangement testing further aiding in confirmation and differential diagnosis. The diagnosis of thyroid IMT requires the integration of multiple immunohistochemical markers. ALK-1 positivity serves as supportive evidence, while positive expression of SMA, MSA, and Vimentin provides essential diagnostic criteria, facilitating differentiation from other tumors originating from fibroblasts or smooth muscle cells, such as solitary fibrous tumor, nodular fasciitis, and malignant peripheral nerve sheath tumor (15, 17).

Previous studies suggest that ALK-positive status may be associated with more aggressive behavior or a higher risk of recurrence. For instance, in one ALK-1-positive thyroid IMT case, the patient had concurrent Hashimoto’s thyroiditis, suggesting that chronic inflammation may contribute to IMT development or progression (12).In another ALK-positive case, the patient underwent partial thyroidectomy and developed metastasis to the right gluteus maximus 17 months post-surgery (9). However, due to the rarity of thyroid IMT, further research is needed to validate these findings. Conversely, ALK-negative IMT may involve other gene rearrangements, such as TFG-ROS1 or ETV6-NTRK3 fusions observed in pulmonary IMT (21). The impact of these abnormalities on thyroid IMT’s biological behavior and prognosis remains unclear.

Given the slow growth, local invasiveness, and low metastatic potential of thyroid IMT, surgery typically achieves curative outcomes and is the primary treatment for localized disease (22). For locally advanced or metastatic cases, comprehensive systemic therapy is recommended. Abnormal ALK expression or structural alterations provide a critical basis for identifying patients who may benefit from ALK inhibitors. Crizotinib and lorlatinib have been used in ALK-positive lung cancer (23, 24) and have shown efficacy in ALK-positive IMT at other sites, whereas ALK fusion-negative patients do not respond to these drugs (25). Although limited cases and the absence of specific clinical trials for thyroid IMT preclude definitive conclusions, the molecular similarities of IMT suggest that ALK-positive thyroid IMT patients may benefit from ALK inhibitors, particularly in cases where surgery is not feasible or recurrence occurs.

In conclusion, this case report documents the identification of the ALK-R395H mutation in thyroid IMT, providing preliminary insights into its molecular characteristics. However, functional studies are needed to elucidate the biological effects of this mutation, including its impact on ALK protein function, signaling pathway activation, tumor proliferation, and invasion. Therefore, the specific mechanisms, prognostic implications, and therapeutic significance of this mutation warrant further investigation.

## Data Availability

The datasets presented in this study can be found in online repositories. The names of the repository/repositories and accession number(s) can be found in the article/**Supplementary Material**.
