# Supplementary material for: Novel ALK gene mutation in inflammatory myofibroblastic tumor of the thyroid: a case report
Source: Front Oncol. 2025 Jun 25;15:1616075. doi: 10.3389/fonc.2025.1616075 (PMC12237923; doi:10.3389/fonc.2025.1616075)
Supplement: Supplementary file 1 [file Table1.docx]

| Supplementary Table 1. List of 66 thyroid-related genes in NGS testing. | | | | | | | | | |
| --- | --- | --- | --- | --- | --- | --- | --- | --- | --- |
| AKT1 | ALK | APC | ATM | BANP | BRAF | CDK12 | CDKN2A | CHEK2 | CTNNB1 |
| DICER1 | EGFR | EIF1AX | EP300 | ERBB4 | EZH1 | FAM193A | FARSB | FGFR1 | FGFR2 |
| FGFR3 | FGFR4 | FLT3 | GLIS3 | GNAQ | GNAS | HRAS | IDH1 | IDH2 | KIT |
| KLK1 | KMT2C | KMT2D | KRAS | LRP1B | MEN1 | MET | MTOR | NCOR2 | NF1 |
| NF2 | NOTCH1 | NRAS | NTRK1 | NTRK3 | PIK3CA | PLEKHS1 | POR | PPARG | PTEN |
| PTH | RB1 | RBM10 | RET | ROS1 | SMAD4 | SPOP | STK11 | SUGCT(C7orf10) | TERT |
| TP53 | TRIM61 | TSC2 | TSHR | VHL | ZNF148 |  |  |  |  |

| Supplementary Table 2. List of 177 thyroid-related fusion genes in NGS testing. | | |
| --- | --- | --- |
| AGGF1(E5)-RAF1(E9) | AGK(E2)-BRAF(E8) | AKAP13(E35)-RET(E12) |
| AKAP9(E21)-BRAF(E10) | AKAP9(E7)-BRAF(E11) | AKAP9(E8)-BRAF(E9) |
| AP3B1(E22)-BRAF(E9) | ATIC(E7)-ALK(E20) | BCL2L11(E3)-BRAF(E10) |
| CARS(E17)-ALK(E20) | CCDC30(E11)-ROS1(E36) | CCDC6(E1)-RET(E12) |
| CCDC6(E2)-RET(E12) | CCDC6(E8)-RET(E11) | CCDC6(E8)-RET(E12) |
| CCNY(E1)-BRAF(E10) | CD74(E6)-ROS1(E32) | CD74(E6)-ROS1(E34) |
| CLTC(E30)-ALK(E20) | CLTC(E31)-ALK(E20) | CREB3L2(E2)-PPARG(E1) |
| DCTN1(E16)-ALK(E20) | DCTN1(E26)-ALK(E20) | EGFR(E24)-RAD51(E4) |
| EGFR(E24)-SEPTIN14(E10) | EGFR(E24)-SEPTIN14(E8) | EML4(E13)-ALK(E20) |
| EML4(E14)-ALK(E20) | EML4(E15)-ALK(E20) | EML4(E17)-ALK(E20) |
| EML4(E18)-ALK(E20) | EML4(E2)-ALK(E20) | EML4(E20)-ALK(E20) |
| EML4(E6)-ALK(E19) | EML4(E6)-ALK(E20) | EML4(E6)-NTRK3(E14) |
| ERC1(E11)-RET(E12) | ERC1(E12)-ROS1(E36) | ETV6(E4)-NTRK3(E14) |
| ETV6(E4)-NTRK3(E15) | ETV6(E5)-NTRK3(E14) | ETV6(E5)-NTRK3(E15) |
| EZR(E11)-ROS1(E34) | FAM114A2(E9)-BRAF(E11) | FGFR1(E1)-PLAG1(E2) |
| FGFR1(E14)-ZNF703(E2) | FGFR1(E17)-TACC1(E7) | FGFR1(E2)-PLAG1(E2) |
| FGFR2(E17)-AFF3(E7) | FGFR2(E17)-BICC1(E10) | FGFR2(E17)-CASP7(E7) |
| FGFR2(E17)-CCAR2(E4) | FGFR2(E17)-CCDC6(E2) | FGFR2(E17)-OFD1(E3) |
| FGFR3(E17)-BAIAP2L1(E2) | FGFR3(E17)-TACC3(E10) | FGFR3(E17)-TACC3(E11) |
| FGFR3(E17)-TACC3(E4) | FGFR3(E17)-TACC3(E8) | FGFR3(E17)-TACC3(E9) |
| FGFR3(E18)-TACC3(E8) | FKBP15(E9)-RET(E12) | FN1(E23)-ALK(E19) |
| GFPT1(E19)-ALK(E20) | GOLGA5(E7)-RET(E12) | GOPC(E4)-ROS1(E36) |
| GOPC(E8)-ROS1(E35) | HIP1(E21)-ALK(E20) | HIP1(E28)-ALK(E20) |
| HIP1(E30)-ALK(E20) | HOOK3(E11)-RET(E12) | IRF2BP2(E1)-NTRK1(E10) |
| KIAA1217(E11)-RET(E11) | KIAA1549(E12)-BRAF(E9) | KIAA1549(E14)-BRAF(E10) |
| KIAA1549(E14)-BRAF(E11) | KIAA1549(E14)-BRAF(E9) | KIAA1549(E15)-BRAF(E10) |
| KIAA1549(E15)-BRAF(E11) | KIAA1549(E15)-BRAF(E9) | KIAA1549(E17)-BRAF(E10) |
| KIAA1549(E18)-BRAF(E9) | KIF5B(E15)-ALK(E19) | KIF5B(E15)-ALK(E20) |
| KIF5B(E15)-RET(E12) | KIF5B(E16)-RET(E12) | KIF5B(E17)-ALK(E20) |
| KIF5B(E22)-RET(E12) | KIF5B(E23)-RET(E12) | KIF5B(E24)-ALK(E20) |
| KIF5B(E24)-MET(E15) | KIF5B(E24)-RET(E11) | KLHL7(E5)-BRAF(E9) |
| KTN1(E29)-RET(E12) | LMNA(E2)-NTRK1(E10) | LRIG3(E16)-ROS1(E35) |
| MACF1(E60)-BRAF(E9) | MKRN1(E4)-BRAF(E10) | MKRN1(E4)-BRAF(E11) |
| MSN(E11)-ALK(E20) | NACC2(E4)-NTRK2(E13) | NCOA4(E7)-RET(E12) |
| NCOA4(E8)-RET(E11) | NCOA4(E8)-RET(E12) | NPM1(E4)-ALK(E20) |
| PAX8(E10)-PPARG(E2) | PAX8(E2)-GLIS1(E2) | PAX8(E2)-GLIS3(E3) |
| PAX8(E7)-PPARG(E2) | PAX8(E8)-PPARG(E2) | PAX8(E9)-PPARG(E2) |
| PCM1(E29)-RET(E12) | POR(E3)-BRAF(E11) | PPFIBP1(E9)-ROS1(E35) |
| PRKAR1A(E7)-RET(E12) | QKI(E6)-NTRK2(E16) | RANBP2(E18)-ALK(E20) |
| RBMS3(E11)-BRAF(E11) | RBPMS(E5)-NTRK3(E14) | RELCH(E10)-RET(E12) |
| RMDN3(E7)-BRAF(E10) | RNF213(E2)-SLC26A11(E8) | SDC4(E2)-ROS1(E32) |
| SDC4(E2)-ROS1(E34) | SDC4(E4)-ROS1(E32) | SEC31A(E20)-ALK(E20) |
| SHTN1(E11)-ROS1(E36) | SLC34A2(E13)-ROS1(E32) | SLC34A2(E4)-ROS1(E32) |
| SND1(E10)-BRAF(E11) | SND1(E10)-BRAF(E9) | SND1(E14)-BRAF(E11) |
| SND1(E14)-BRAF(E9) | SND1(E9)-BRAF(E9) | SPECC1L(E10)-RET(E12) |
| SQSTM1(E5)-ALK(E20) | SQSTM1(E5)-NTRK1(E10) | SQSTM1(E5)-NTRK3(E14) |
| SSBP2(E12)-NTRK1(E12) | STRN(E3)-ALK(E20) | SYN2(E5)-PPARG(E2) |
| TANK(E4)-BRAF(E9) | TBL1XR1(E9)-RET(E12) | TFG(E4)-ALK(E20) |
| TFG(E4)-RET(E11) | TFG(E5)-ALK(E20) | TFG(E5)-MET(E15) |
| TFG(E5)-NTRK1(E9) | TFG(E5)-ROS1(E35) | TFG(E6)-ALK(E20) |
| THADA(E28)-LOC389473 | THADA(E29)-LOC389473 | THADA(E30)-IGF2BP3(E3) |
| THADA(E30)-LOC389473 | THADA(E31)-IGF2BP3(E2) | THADA(E31)-LOC389473 |
| THADA(E35)-IGF2BP3(E2) | THADA(E35)-IGF2BP3(E3) | THADA(E36)-LOC389473() |
| TPM3(E7)-ALK(E20) | TPM3(E7)-NTRK1(E10) | TPM3(E8)-ROS1(E35) |
| TPR(E15)-ALK(E20) | TPR(E21)-NTRK1(E10) | TPR(E6)-NTRK1(E12) |
| TRIM24(E10)-BRAF(E9) | TRIM24(E3)-BRAF(E10) | TRIM24(E5)-BRAF(E8) |
| TRIM24(E8)-BRAF(E11) | TRIM24(E9)-BRAF(E9) | TRIM24(E9)-RET(E12) |
| TRIM27(E3)-RET(E12) | TRIM33(E16)-NTRK1(E12) | TRIM33(E16)-RET(E12) |
| UACA(E17)-LTK(E10) | VCL(E16)-ALK(E20) | ZC3HAV1(E7)-BRAF(E11) |
